# A comparative review of myocarditis in pediatrics versus adults: pathogenesis, diagnosis, and management

**DOI:** 10.3389/fimmu.2025.1601307

**Published:** 2025-09-26

**Authors:** Jacob C. Ricci, Nick A. Farahani, Cormac J. Davis, Kyra G. Ritter, Lauren M. Parrow, Priya I. Tomerlin, Ashley A. Darakjian, Katherine Gegoutchadze, Dipankar Gupta, Katelyn A. Bruno

**Affiliations:** ^1^ Division of Cardiovascular Medicine, Department of Medicine, University of Florida, Gainesville, FL, United States; ^2^ Biomedical Sciences Program, University of Florida, College of Medicine, Gainesville, FL, United States; ^3^ Center for Integrative Cardiovascular and Metabolic Disease, University of Florida, Gainesville, FL, United States; ^4^ Department of Physiology and Aging, University of Florida, Gainesville, FL, United States; ^5^ Department of Pharmacology and Therapeutics, University of Florida, Gainesville, FL, United States; ^6^ Congenital Heart Center, College of Medicine, University of Florida, Gainesville, FL, United States; ^7^ Department of Pediatrics, University of Florida, Gainesville, FL, United States

**Keywords:** myocarditis, dilated cardiomyopathy, pediatrics, coxsackievirus, age differences

## Abstract

Myocarditis is an inflammatory heart disease that is more prevalent in men. The etiology of myocarditis is often multifactorial with viral infections being a predominant cause of myocarditis. Other etiologies such as autoimmune mediated or secondary to certain medical therapies such as immune checkpoint inhibitors are also seen however less commonly. The wide spectrum of clinical symptoms with which these patients present and the lack of reliable patterns or biomarkers of progression make it difficult to both diagnose and risk-stratify patients. Importantly, this disease is widely prevalent in pediatric populations and is a leading cause of sudden cardiac death in young patients. However, much of the knowledge of pathogenesis and treatment of this disease is extrapolated from adult studies. Current research in myocarditis has increasingly identified the role of hormones and the apparent sex differences seen predominantly in adult patients; however, such data is not well established in pediatric patients. Thus, there is an increased need to evaluate the age and sex-based differences in pediatric patients with myocarditis. Therefore, this review aims to present an overview of our current understanding of pathogenesis, diagnosis, and treatment strategies for myocarditis, with an emphasis on outlining both adult and pediatric studies to emphasize the continued need for research into this disease.

## 1 Introduction

Myocarditis is caused by acute or chronic inflammation of the cardiomyocytes, leading to myocardial edema, injury, and necrosis (1). There have been ongoing efforts to define myocarditis better, and the most recent AHA guidelines suggest four strata: biopsy-proven, cardiac magnetic resonance imaging (CMR)-confirmed clinically suspected, clinically suspected, and possible myocarditis (2). The pathophysiology of myocarditis is multifactorial; however, it occurs primarily due to viral infections, though other pathogens and hypersensitivity reactions are also other known triggers (3). Serology can be utilized to identify viruses that could be causative for myocarditis but it is well accepted that a definitive diagnosis of viral caused myocarditis can only be made via positive PCR on the endomyocardial biopsy. Clinically, patients have heterogeneous presentations ranging from mild symptoms such as non-specific chest pain to heart failure, life-threatening arrhythmias, and sudden cardiac death (4, 5). The natural history after the episode of acute inflammation includes the possibility of complete resolution of clinical symptoms, improvement of myocardial dysfunction, development of dilated cardiomyopathy, and need for heart transplantation or death (4–8).

The true prevalence of myocarditis is difficult to estimate due to inconsistency in definition, heterogeneous presentation, and often sub-clinical self-limiting illness. The Global Burden of Disease Study estimated the number of myocarditis cases using discharge records to be roughly 22 for every 100,000 patients (9). More recent estimates range from 10.2 to 105.6 per 100,000 worldwide, with an annual occurrence of around 1.8 million cases (10). It has been well documented that men are at two-to-four-fold increased risk of developing myocarditis compared to females (11–21). Additionally, it has been shown that males who develop myocarditis are also at an increased risk of progressing to dilated cardiomyopathy (22). Overall, the sex differences in the incidence and natural history of myocarditis have led to significant research evaluating the impact of sex hormones on the inflammatory response in myocarditis. Most studies discussing these sex differences have been conducted in adult patients. However, there is data in younger patients that points to male predominance and even an age-related increase in predominance of males with myocarditis (23, 24). Conversely, other studies have shown no significant difference in the proportion of males versus females with myocarditis (25). A recent study of over 15,000 patients with myocarditis showed that this sex ratio was consistent in their adult cohort but was not present among pediatric patients (26).

Although several studies have documented the incidence and outcomes of viral myocarditis in children, there is a paucity of research comparing the unique differences in pediatric versus adult patients with myocarditis. Viral myocarditis is more often seen in children than in adults, and children are more likely to present with acute myocarditis (sudden onset) rather than chronic myocarditis, which is typically seen in adults. Also, the incidence of myocarditis in children varies with age, having a bimodal distribution, being higher in infants and rising again in young adults. While there are fundamental clinical differences between these two patient populations, there is a need for foundational basic, translational, and clinical research specifically focusing on myocarditis in the pediatric population to further our understanding. Therefore, this paper aims to review the pathogenesis, diagnosis, and treatment of viral myocarditis, highlighting our current understanding of the differences between pediatric and adult populations.

## 2 Pathogenesis

**Figure 1:**
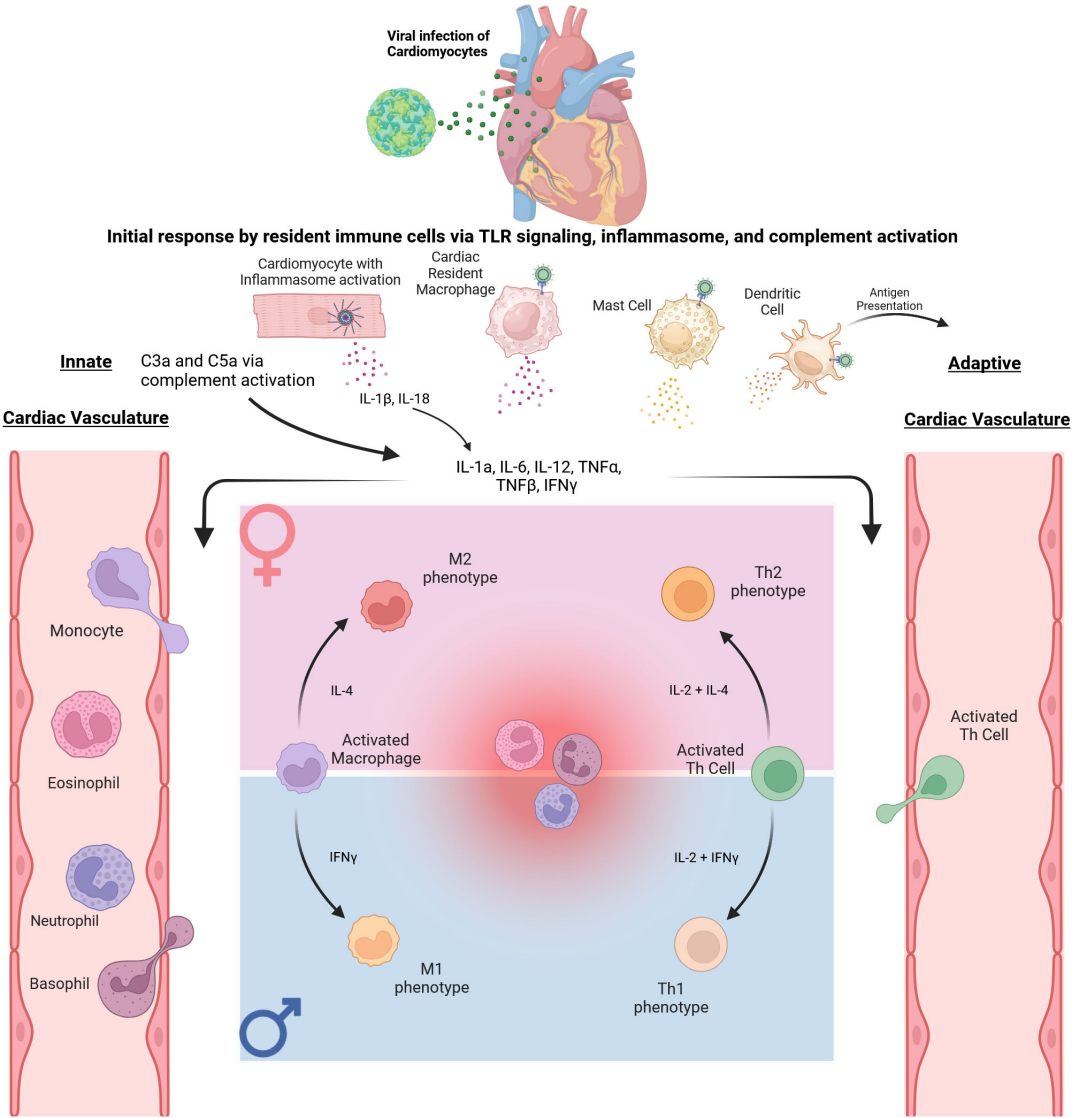
Immune response to Coxsackievirus B3 in Cardiac Tissue. The initial immune response begins with CVB3 trafficking to the heart. The initial infection of cardiomyocytes and resident immune cells triggers an innate inflammatory response via signaling through TLR 2, 4, and 7, as well as complement and inflammasome activation, ultimately leading to secretion of proinflammatory cytokines IL-1β, IL-18, IL-1α, IL-6, TNFα, TNFβ, and IFNγ. These cytokines promote extravasation of innate immune cells from the vasculature to the site of inflammation. Specific sex differences within this process are displayed via the pink and blue panels. In particular, as monocytes exit the vasculature and differentiate into macrophages, it has been shown that an M2 polarization may be favored in female mice. In contrast, an M1 polarization may be more prominent in male mice. As the immune response progresses, dendritic cells can migrate and present antigens to Naïve T cells. These subsequently activated T cells then migrate to the site of inflammation. Subsequent differentiation of activated CD4+ Th cells also displays sex differences as immune cells display androgen and estrogen receptors. An example is shown in the figure with CD4+ Th cells differentiating to the Th2 subtype in females while differentiating to the Th1 subtype in males.

While numerous viruses have been associated with myocarditis, including parvovirus B19, human herpesvirus 6, and adenovirus, one of the most common viral agents known to cause myocarditis is coxsackievirus B (CVB) (27, 28). CVB is a positive single-stranded RNA belonging to the enterovirus group within the *Picornaviridae* family (29). Like other enteroviruses, CVB is transmitted via a fecal-oral route, and initial infection is through the GI tract, though viral kinetic experiments have shown that replication occurs in the heart, spleen, thymus, and pancreas (30). The pathogenesis of myocarditis is still being investigated, and clinical samples and varied mouse models are being utilized to better understand disease development and progression. Some of the known immune cell involvement in viral myocarditis are illustrated in **Figure 1**.

Other etiologies for myocarditis include various parasites and bacteria (i.e. Trypanosoma Cruzi, Staphylococcus) (31, 32). Furthermore, myocarditis has also been reported as a side effect of many medications and vaccines, most recently the COVID-19 vaccine (33). Medication classes including antipsychotics (clozapine) and salicylates (mesalazine) have been associated with myocarditis (34). Additionally, though rare, myocarditis secondary to the use of immune checkpoint inhibitors has also been reported (35, 36). Immune checkpoint inhibitor medications, primarily those targeting programmed death (PD)-1, programmed death ligand (PDL)-1, and cytotoxic T-lymphocyte antigen (CTLA)-4, are widely used in the treatment of certain cancers. Though their mechanism of action is to primarily block these targets from suppressing the immune system, they can lead to cardiac toxicity through infiltration of multiple types of immune cells including CD8+ T cells, and CCR2+ macrophages (37–39).

Though viral etiologies are often identified as the most common cause of myocarditis in North America (40, 41), many autoimmune diseases also have cardiac manifestations, including myocarditis. A recent systematic review found that myocarditis is prevalent within a wide spectrum of autoimmune diseases such as cardiac sarcoidosis, systemic lupus erythematosus, systemic sclerosis, eosinophilic granulomatosis with polyangiitis, demato/polymyositis, and Takayasu’s arteritis. Interestingly, the presence of biopsy confirmed myocarditis in these diseases has been shown to be associated with a worse prognosis (42). Finally, although an area of ongoing research, genetic evidence gathered from familial cases of recurrent myocarditis and pediatric myocarditis support the idea of an underlying genetic susceptibility (43, 44). A recent study of 336 patient with myocarditis found that, compared to healthy controls, there was an increased enrichment of truncating variants of cardiomyopathy associated genes. In particular, truncated variants in the desmoplakin gene (DSP) were found in myocarditis patients with normal LV function and ventricular arrhythmia while truncated variants in the Titin gene (TTN) were found in myocarditis patients with reduced ejection fraction (45). Though more research is needed into the genetic aspects of myocarditis, it is an etiology that should be considered clinically.

Despite the varied etiologies, viral infections continue to be the predominant cause leading to myocarditis with differential impacts on various ages and varied clinical presentations.

### 2.1 Viral entry

Mechanisms of entry utilized by these viruses are multi-faceted and often strain-dependent. Usually, the virus gains entry to cardiomyocytes, endothelial cells, and stromal cells using virus-specific receptors. The coxsackie-adenovirus receptor (CAR) is a highly conserved, widely distributed transmembrane protein that CVB utilizes to aid in viral attachment as well as infection (46, 47). However, CAR has been shown to localize to tight junctions in polarized epithelial cells. Therefore, the mechanism of viral access to this receptor has been an ongoing topic of interest (48). Decay Accelerating Factor (DAF or CD55) is a member of the complement control family. It is an additional receptor for many of the enteroviruses, though, compared to CAR, the mode of binding is less conserved (49–51). However, DAF plays a critical role in viral infection via CAR. Viral binding to DAF on the apical cell surface has been shown to trigger a signaling cascade, ultimately leading to Rac-dependent actin rearrangements (52). These rearrangements allow for the delivery of the virus to the tight junctions where interactions with CAR can occur.

### 2.2 Immune response

Focusing on CVB infection and immune response in the heart, during the initial phase of acute viremia, there is a near immediate response from resident cells in the myocardium, including increased expression of IL-1α, IL-1β, IL-6, IL-18, TNFα, TNFβ, and IFNγ (53, 54). This initial response is predominantly due to the presence and activation of Toll-like Receptors (TLRs) on the cell surface and cytosol. While the role of TLRs in CVB immune response still needs better understanding, three classes of TLRs are integral to the initial immune response: TLR 2, 4, and 7 (55, 56). Activating these receptors provides chemotactic signaling for other innate immune cells and creates a cytokine profile that impacts eventual adaptive immune response.

As viral replication and early inflammatory response progress, cells of the innate immune system, particularly Natural Killer (NK) cells, and macrophages, are recruited to the site of damage (57). While both cell types are key to the innate immune response and controlling viral replication, macrophages also present a unique response to infection in their ability to polarize (58, 59). In response to specific cytokine profiles, macrophages can either take on a proinflammatory M1 or anti-inflammatory M2 polarization (60, 61). Interestingly, sex differences in polarization exist in CVB myocarditis, with males having a predominant M1 profile compared to females, who tend to develop an M2 profile (62, 63). Though sex hormones have been shown to impact this polarization process (64), this effect has been analyzed primarily in adult mice, and the role of age has not been investigated.

With innate immune cell infiltration, there is an accompanying increase in pro-inflammatory cytokine levels and eventual activation of the adaptive immune system via antigen presentation on Major Histocompatibility Complexes (MHCs) to naïve CD4+ Th cells (65). This antigen presentation, combined with specific cytokine profiles, leads to the differentiation of activated CD4+ T-cells into four possible subtypes of helper T cells: Th1, Th2, Th17, or regulatory T cells (Treg). Th1 is involved with responses to intracellular pathogens by expressing IFNγ and TNFα, while Th2 and Th17 cells are primarily involved in extracellular pathogen responses expressing IL-6, IL-4 and IL-10 and IL-17, IL-22, and IL-21, respectively (66, 67). In contrast, Treg cells primarily aim to control the immune response and maintain homeostasis (68). While this process of differentiation is well reviewed in response to CVB (69), there are notable sex differences in the ratios of these subtypes, as estrogen and androgen receptors are present in many immune cells, including T cells (70). Specifically, it has been shown that estrogen favors a Th2 response and promotes IL-4 and IL-10 expression while also inhibiting the Th1 response (71–75). Conversely, testosterone leads to a predominant Th1 response, though there is evidence that inflammation in males is attributed more to innate rather than adaptive cells (76, 77).

### 2.3 Role of aging

The immune system constantly evolves throughout our lives, beginning in infancy, peaking near puberty, and subsequently declining with age (78). Given this evolution, the impact of sex hormones on the immune system has been a significant research topic and has been well-reviewed (79). Moreover, the interplay between sex hormones and the immune system in the context of CVB myocarditis is well documented and does support the sex differences seen in adult patients.

However, while puberty may be a convenient dividing line, sexual dimorphisms in the immune system may be present before the onset of puberty. Rosen et al. analyzed age and sex differences in mice both pre- and post-puberty. Interestingly, they found that in 3-week-old mice, mitogenic response to ionomycin and phorbol ester was more significant in female splenocytes than male splenocytes. Yet, in mice 4–6 weeks of age, this was reversed, with males displaying a more substantial response (80). Additionally, Castro demonstrated that prepubertal orchiectomy in mice resulted in relative thymic hyperplasia compared to controls (81). Overall, future work should include an investigation into the prepubertal immune response, particularly with respect to CVB myocarditis.

## 3 Diagnosis

Diagnosis of myocarditis presents a significant difficulty as patients may present with a wide array of symptoms ranging from mild viral illness to new onset heart failure, and even with evidence of ST-elevation myocardial infarction (STEMI) (82–85). Currently, the gold standard diagnostic test for myocarditis is endomyocardial biopsy. While recent large-scale prospective studies have shown that EMB is safe and prognostically valuable in both adults and children, the United States tends to trail behind Europe (86–88). In 2021, a joint position statement from the American and Japanese Heart Failure Societies outlined potential clinical indications for EMB, including suspected acute myocarditis with acute heart failure or suspected myocarditis in a hemodynamically stable patient (89). In 2024, the American College of Cardiology (ACC) released their consensus decision pathway on the diagnosis and management of myocarditis and recommended that EMB be performed in “clinical scenarios where the prognostic and diagnostic value of the information gained outweigh the procedural risks” (90). Thus, while no “one-size-fits-all” test can provide a definitive diagnosis, currently available testing methods can help the medical team differentiate myocarditis from other cardiac pathologies.

### 3.1 Clinical presentation

Clinical presentation of acute myocarditis is heterogeneous, ranging from non-specific chest pain to cardiogenic shock. In **Table 1** we have summarized various presenting features described in the literature as the presenting symptoms in pediatric and adult patients. These symptoms are not an exhaustive list, and many patients have overlapping clinical features. Additionally, patients may present with any combination of these symptoms (i.e., adult patients often do not present with fever or constitutional symptoms). In adult patients with myocarditis, chronic presentations may also be seen with cardiac symptoms such as chest pain, often with preserved systolic function and histologic evidence of persistent myocardial inflammation. The pediatric population usually will have a history of a viral prodrome in about two-thirds of patients, arrhythmias in about 45%, syncope in 10%, and occasionally may present as sudden cardiac death. The clinical symptoms may overlap in adults and pediatric patients, with fatigue, shortness of breath, fever, chest pain, dyspnea, and cough being common presenting signs. The patient usually may demonstrate subtle signs of heart failure to cardiogenic shock depending on the severity of the presentation. Especially in pediatric patients the symptoms differ among age groups. For example, infants with myocarditis may present with persistent tachypnea, poor feeding, getting tired with feeding, recurrent respiratory infections, diaphoresis and/or other overt signs of heart failure. However, an adolescent may have a presentation similar to a young adult with fatigue, shortness of breath, tiredness, poor appetite or abdominal pain especially with severely reduced ventricular function. Other presentations include acute coronary syndrome (more common in adults) and new or worsening heart failure symptoms. Subacute or chronic myocarditis, seen more commonly in adults, usually presents with an indistinct onset of illness (generally more than three months), which has been described as either chronic active or chronic persistent myocarditis. On the other hand, pediatric patients may be more likely to present with fulminant disease (2). Regardless, in patients with clinical suspicion, further evaluation is warranted using laboratory studies and other biomarkers to diagnose myocarditis further.

**Table 1:** Common presenting symptoms of myocarditis between children and adults.

	Pediatric	Adult	Reference
*Constitutional*			
*Fever*	x	x	(138, 185–193)
*Fatigue*	x	x	(187–190, 192, 194, 195)
*Diaphoresis*	x	x	(187, 192)
*Poor feeding*	x		(138, 196)
*Myalgias*	x		(188–190)
*Decreased exercise tolerance*	x		(192)
*Failure to thrive*	x		(185)
*Lethargy*	x		(192, 196)
*Chills*	x	x	(187, 190)
*Head, Ears, Eyes, Nose, and Throat*			
*Headache*	x	x	(187, 189, 190)
*Sore throat*	x		(190)
*Rhinorrhea*	x		(192)
*Dizziness*	x		(195)
*Respiratory*			
*Cough*	x	x	(186, 192–194, 196)
*Dyspnea*	x	x	(138, 185, 187, 188, 190, 192, 194–198)
*Tachypnea*	x		(192)
*Wheezing*	x		(192)
*Apnea*	x		(192)
*Respiratory Distress*	x		(196, 199)
*Cyanosis*	x		(192)
*Cardiovascular*			
*Chest Pain*	x	x	(138, 186–190, 192, 195, 197, 198, 200–202)
*Palpitations*	x	x	(185, 188, 192, 195, 197, 198, 203, 204)
*Syncope*	x	x	(190, 192, 198, 200)
*Shock*	x	x	(185, 188, 198, 200, 202, 203)
*Heart Failure*	x	x	(138, 185, 187, 199, 200, 204)
*Abdominal*			
*Abdominal pain*	x		(192, 193, 196)
*Nausea/Emesis*	x	x	(187, 190, 192, 193, 196)
*Diarrhea*	x		(191, 193)
*Hepatomegaly*	x		(185, 192)
*Neurologic*			
*Numbness/Tingling*	x		(190)
*Urinary*			
*Decreased urine output*	x		(192)

### 3.2 Laboratory evaluation

According to the 2024 ACC and AHA expert consensus documents on the diagnosis and management of myocarditis, initial laboratory evaluation should include CBC with differential, biomarkers of cardiac necrosis such as high-sensitivity troponin and creatinine kinase-MB and nonspecific inflammatory biomarkers such as C-reactive protein and erythrocyte sedimentation rates should be obtained (90, 91). Viral swabs and antibodies are also obtained to ascertain the etiology of the myocarditis. There continues to be a lack of universal diagnostic criteria for myocarditis and heterogeneity of clinical presentation makes it even more challenging to compare various studies. Generally, these initial evaluations are mirrored in the recommendations for pediatric populations though disease specific guidelines are not directly published (92). For both populations, there is support for genetic evaluation. However, genetic evaluation is more common in children and often screens for genes in which there is definitive evidence of mutations causing cardiac disease while in adults there may be more focus on genetic variants that are overrepresented in acute myocarditis populations (93, 94).

While there are no definitive laboratory markers for the diagnosis of myocarditis, ongoing developments in the area of acute phase reactants and markers of inflammation or damage have shown promise. A study of 44 patients with confirmed myocarditis showed that myoglobin levels in peripheral blood positively correlated with disease severity. Upon further testing, it was shown that, with a cutoff level of ≥ 87 μg/L, myoglobin levels were able to identify mice with myocarditis with a sensitivity and specificity of 92% and 80%, respectively (95). Recently, Villatore et al. showed that pentraxin 3, an acute phase protein, was detected in the plasma of 86.7% (26/30) myocarditis patients compared to 0% (0/10) healthy controls. Additionally, pentraxin 3 detection was performed in cardiac samples from all patients, but they noted that higher signal intensity was associated with viral myocarditis compared to autoimmune myocarditis (96). MicroRNA has also been shown to be helpful in the diagnosis of myocarditis. Specifically, a microRNA (mmu-miR-721), which has a human homolog (hsa-miR-Chr8:96), is synthesized by Th17 cells. Using this microRNA, patients with myocarditis could be distinguished from those with myocardial infarction with an AUROC of 0.927 (0.879-0.975) (97). Serum Anti-heart and Antinuclear antibodies have also been shown to be predictive of immunosuppressive treatment response in biopsy proven myocarditis. However, this study only considered autoimmune etiologies (98). Despite this, there is evidence that autoantibodies may be a valuable marker in viral myocarditis. In particular, it has been shown that autoantibodies are produced in response to cardiac injury during coxsackievirus infection (99). Furthermore, anti-myosin antibodies identified in myocarditis patients were found to bind to β-adrenergic receptors on the heart thus impacting heart rate and contractility (100). Nevertheless, the role of autoantibodies in the pathogenesis and progression of myocarditis remains unclear and should be a focus of research moving forward (101).

Finally, serum sST2 (also known as interleukin-1 receptor-like 1) may be a valuable biomarker in differentiating myocarditis. The Fairweather group has shown that, within a subset of patients with clinically suspected or biopsy-confirmed myocarditis, elevated sST2 levels were associated with increased risk of heart failure based on New York Heart Association (NYHA) class in men under 50 years of age (13). Interestingly, though sera sST2 has been shown to be elevated in Cardiovascular Disease (CVD) compared to healthy controls, sex differences are present for subsets with myocarditis and cardiomyopathy. Notably, in patients under 50 years of age with myocarditis, males have a significantly higher sera sST2 level compared to females. However, this sex difference is not present in myocarditis patients greater than 50 years of age (102). Two studies have assessed the value of sST2 as a biomarker for myocarditis in children, although the focus was on fulminant myocarditis. One retrospective study of 203 patients under 11 years of age found that elevated levels of sST2 correlated with fulminant myocarditis, while another study of 144 patients under 14 years of age showed that, with a cut-off value of 63.8 ng/mL, sST2 had a sensitivity and specificity of 84.13% and 88.9%, respectively (103, 104). Ultimately, further research is needed into potential biomarkers for myocarditis with specific attention to the possible impacts of sex as well as age.

An electrocardiogram (EKG) is a valuable diagnostic tool; however, in myocarditis, it has been shown to lack specificity (105). Chen et al. reported that among adult patients admitted with a diagnosis of acute myocarditis, a wide QRS-T angle (>100°) was present in 13% of patients and was a predictor of increased morbidity and mortality (106). Like acute myocardial infarction, myocarditis may be associated with regional ST elevations and Q waves. Some studies have shown that the presence of Q waves or left bundle branch block was associated with higher rates of death or transplantation (107) while other studies have not been able to confirm these findings (108, 109).

Although not specific to CVB myocarditis, utilizing lab values and EKG findings to monitor and predict disease progression has been a clinical research focus, particularly during the COVID-19 pandemic. Patel et al. found that among 99 patients with a median age of 3 years old (0–22) who were hospitalized for symptomatic heart failure, lymphocytopenia, hyponatremia, and elevated serum creatinine were independent predictors for risk of death or need for a heart transplant (110). Regarding electrocardiographic findings, children with DCM who have increased P maximum, P distribution, QT dispersion, QTc maximum, QTc dispersion, Tp-emax, Tp-e dispersion, or QRS duration had an increased risk of death (111). However, specific studies designed for the comparison of EKG findings between pediatric and adult populations with myocarditis remain to be conducted.

### 3.3 Echocardiography

Echocardiography is an integral tool in cardiac evaluation for myocarditis within both pediatric and adult populations (90–92). Echocardiographic findings may vary from mild to severe ventricular dysfunction with or without evidence of ventricular dilation and/or wall motion abnormalities. However, conventional 2D echocardiography lacks the ability to detect potential deformations or changes in cardiac function in all but severe cases of myocarditis (112, 113). Though its utility in diagnosing acute myocarditis is variable, echocardiography has a role in monitoring patients, particularly children, who have a history of myocarditis and dilated cardiomyopathy (DCM). Decreased ejection fraction, myocardial performance index, and increased left ventricular end-systolic and end-diastolic dimensions (LVESD and LVEDD, respectively) have been shown to be associated with an increased risk of death or transplant in pediatric patients (111). The transplant/mortality likelihood increased by 41.6% and 19.8% for each unit increase in LVEDD and LVESD z scores, respectively (80). Ryo et al. also showed that increased left ventricular ejection fraction (LVEF), mitral E-deceleration time, right ventricular (RV) fractional area change, and tricuspid annular plane systolic excursion were associated with reduced mortality and transplant risk (114).

More recently, there has been increased interest in the changes in myocardial strain with myocarditis. Strain, or deformation, imaging allows for quantifying regional myocardial function, yet its adoption has been slow in clinical echocardiography, which primarily utilizes visual assessment of wall motion (115–118). Fairweather et al. showed in their CVB murine model that there was a significant reduction in global longitudinal strain in infected mice compared to healthy controls as well as sex differences, with males having a more severe reduction compared to females (113). In humans, global longitudinal peak systolic strain, as well as global circumferential strain, were lower in patients with acute myocarditis, and a multiparametric model including epicardial left ventricular global longitudinal strain was able to identify patients who were subsequently diagnosed with myocarditis correctly (119, 120).

Use of strain calculations for acute viral myocarditis in pediatric patients is limited, though one study from Gursu et al. showed strain values were significantly reduced in patients (mean age 12.3 years) compared to healthy age-matched controls (112). Other studies of strain in pediatric patients have focused on multisystem inflammatory syndrome in response to COVID-19 but show similar effects (121, 122).

### 3.4 Cardiac magnetic resonance

While endomyocardial biopsy remains the gold standard for diagnosing myocarditis, Cardiac magnetic resonance (CMR) is increasingly being used for non-invasive diagnosis. Due to its non-invasive nature, it is recommended over endomyocardial biopsy except in certain specific scenarios (90). CMR imaging enables the detection of various features of myocarditis, including evidence of inflammatory hyperemia and edema, late gadolinium enhancement (LGE) suggestive of myocyte necrosis and scar, changes in ventricular size and geometry, regional and global wall motion abnormalities, and identification of accompanying pericardial effusion. Myocardial inflammation is diagnosed on CMR via the Lake Louise Criteria based on the following criteria: myocardial edema on T2 mapping (demonstrated by regional or global increase of either native T2 times or T2 signal intensity) or non-ischemic myocardial injury (demonstrated by regional or global increase of either native T1 time, extracellular volume, or a regional increase in late gadolinium enhancement (LGE)) (123). Overall, CMR data has been highly effective in diagnosing myocarditis and providing valuable prognostic indicators and risk-stratifying patients (124–127).

However, there are potential limitations in terms of sensitivity. Mainly, CMR has been shown to have high diagnostic sensitivity for myocarditis in patients with an “infarct-like” (fever, chest pain, troponinemia, and ST changes) pattern compared those with “cardiomyopathic” (LV dysfunction with no EKG changes) or “arrhythmic” (acute onset of life-threatening arrhythmia) patterns (128). Moreover, this difference in sensitivity has also been shown to persist even after use of parametric tissue mapping as recommended by the updated Lake Louise Criteria (129).

Finally, it is important to consider that there is evidence of reduced diagnostic sensitivity beyond 8 weeks from symptom onset (130) and the baseline sex-related differences in the patient population, especially adolescents (131). Moreover, performing CMR in younger patients can be difficult as they may have difficulty following breathing instructions or may potentially be too sick to undergo anesthesia safely (132).

### 3.5 Other diagnostic tools

Cardiac catheterization with coronary angiography may be used in select patients presenting with acute coronary syndrome or in those with high-risk features for ischemic heart disease on noninvasive testing. While not widely used clinically, there has been additional research into other diagnostic tools for diagnosing myocarditis. Lekakis et al. in 1995 showed that Indium-111 monoclonal anti-myosin antibody uptake ratios between the heart and lung were significantly higher than controls (133). Additionally, using 18F-FDG PET may be beneficial in diagnosing myocarditis, particularly if arrhythmias are present or if the time since symptom onset is more prolonged. However, prospective trials are still necessary (134, 135). In mice, it has been shown that translocator protein 18kDa (TSPO) is upregulated in male mice on CD11-b(+) immune cells, and this upregulation translates to significantly higher uptake of [ (125)I]-IodoDPA-713 on microSPECT in CVB3 infected males compared to uninfected controls (136). The use of TSPO in diagnosing myocarditis patients has not been translated to human patients at this time.

An additional imaging modality that has the potential to aid clinicians in diagnosing myocarditis is magnetocardiography (MCG). While MCG has been present since the 1960s, it has primarily been researched in the setting of ischemic heart disease. Still, its use has more recently extended into myocarditis and inflammatory cardiomyopathies. MCG relies on detecting the magnetic field generated by the physiological current that passes through the heart with each cycle. Using this technology, it has been shown that MCG was able to not only differentiate those patients with cardiomyopathy from those without but also showed improving values 7 days after treatment initiation compared to 30 days when echo was used (137).

Finally, genetic evaluation may have a role. A recent study published the results of forty-two pediatric patients with biopsy-proven myocarditis who underwent genetic screening. Among those patients who progressed to DCM at a faster rate, potential pathogenic variants in *BAG3*, *DSP*, *LMNA*, *MYH7*, *TNNI3*, *TNNT2*, and *TTN* were seen (138). Therefore, there may still be value in genetic testing patients with newly diagnosed myocarditis to predict which patients may be more likely to progress to DCM. Overall, while there has been considerable research into the diagnosis of myocarditis, research into the differences between pediatric and adult populations remains sparse. Thus, future work to better understand these differences may be beneficial for accurately diagnosing myocarditis within each of these populations.

## 4 Treatment

Treatment for myocarditis remains a significant therapeutic challenge due to the lack of specific targeted therapies. Current treatment guidelines primarily focus on symptomatic relief and treatment of any associated heart failure symptoms in line with heart failure guidelines. There may also be a role for anti-inflammatory medications use which function to decrease the body’s inflammatory response as well as immunosuppressive therapies which suppress the body’s immune response. However, while there is agreement on the use of immunosuppressive medications in specific noninfectious presentations, there remains disagreement on the utility of immunosuppression in lymphocytic myocarditis in which viral PCR of the EMB sample is negative. However, intravenous immunoglobulin (IVIG) may be considered in inflammatory conditions (90). Pocapavir is an investigational enteroviral capsid inhibitor that has shown promise in a limited number of cases of severe neonatal enterovirus myocarditis. At this time, there are no disease-specific approved antiviral therapies for patients with myocarditis (139, 140). However, the JACC 2024 consensus statement on myocarditis notes that these medications may be an acceptable alternative though recommends consultation with an infectious disease expert (90). Overall, this highlights the need for robust prospective clinical trials and further research into treatment strategies specific to myocarditis.

### 4.1 Immunosuppressive and anti-inflammatory therapies

Corticosteroids have been extensively studied for their role in managing viral myocarditis. A landmark trial by Mason et al. failed to show a significant improvement in LVEF with the use of corticosteroids with either cyclosporine or azathioprine (141). A 2013 Cochrane meta-analysis of eight randomized controlled trials (RCTs) involving 719 participants revealed mixed outcomes (142). Of these eight trials, two focused specifically on pediatric populations. The analysis showed no significant reduction in mortality or rates of death/heart transplant and no improvement in the NYHA class. However, short-term improvements in LVEF at 1–3 months were observed with corticosteroids. This trend continued when the pediatric studies were analyzed separately. Long-term improvements in LVEF and decreased levels of biomarkers such as CK-MB and α-HBDH were also noted, though these results were only reported in adult studies. Notably, a 2010 study involving 68 children (mean age 3.7 ± 2.9 years) demonstrated that prednisolone significantly improved LVEF, with more children achieving over 60% LVEF compared to the control group (143). Another study in pediatric patients demonstrated that high-dose steroids in conjunction with IVIG to treat acute myocarditis can be safe without significant infections or long-term side effects. However, there still is a need for a prospective comparison of a combination of high-dose steroids with IVIG versus other therapies (144). A Taiwanese study showed that high-dose steroid or IVIG therapy had no significant effects on major in-hospital complications, late heart failure hospitalization, and long-term mortality (145). These conflicting results highlight the need for RCTs to answer these questions and the ongoing need for an enhanced understanding of the pathogenesis to develop targeted therapies.

A 2021 study investigating colchicine’s efficacy in myocarditis, as determined by cardiac MRI (CMR) criteria, enrolled 146 participants, with eighty-six receiving colchicine. The results were promising, as 64% of patients treated with colchicine achieved complete resolution of myocarditis, compared to only 19% in the control group (146). Biopsy samples of patients with myocarditis demonstrate an amplified expression of NLRP3 inflammasome and related cytokines including interleukin (IL)-1β and IL-18, reflecting greater myocardial injury. Colchicine targets these pathways by reducing superoxide production and inhibition of inflammasomes and IL-1β production. Additionally, colchicine has shown to have antifibrotic and endothelial-protective features. Overall, colchicine may be useful in patients with myocarditis by targeting the underlying inflammatory processes however further studies with larger cohorts are warranted.

Anakinra, an interleukin-1 receptor antagonist, was investigated in the ARAMIS trial (Anakinra Versus Placebo for the Treatment of Acute Myocarditis; NCT03018834), which is the largest randomized trial in myocarditis to date. This double-blind RCT enrolled 120 symptomatic patients to evaluate the efficacy of Anakinra in mitigating the inflammatory cascade associated with acute myocarditis. They found that patients receiving a short course of Anakinra did not have a significantly increased number of days free of myocarditis complications (147). There was also a trial of this agent in pericarditis during the COVID-19 pandemic, but it was terminated early due to drastic benefits seen within the first 24 hours of administration (148). However, the FDA has not yet approved Anakinra for use in either indication.

A 2020 study evaluating immunosuppressive therapy in acute inflammatory cardiomyopathy showed that immunosuppressive therapy did not significantly improve outcomes compared to those who did not receive it. However, patients who did not receive the therapy had higher rates of poor lymphocytic infiltrates at endomyocardial biopsy, suggesting potential differences in disease pathology (149).

Finally, a recent study was published that evaluated the use of individualized prednisone and azathioprine dosing strategies, compared to optimal medical therapy for heart failure, in biopsy proven myocarditis (150). This study was an extension of the TIMIC trial which showed potential efficacy of these medications in virus-negative inflammatory cardiomyopathy and at prespecified doses and durations (151). Among 91 patients receiving tailored immunosuppressive therapy, compared to the 267 receiving the optimal therapy, it was shown that, at baseline, patients in the immunosuppressive groups tended to have lower cardiac ejection fraction at baseline. At 5-year follow-up, there was no significant difference in overall survival between the two groups. However, it should be noted that this study included all etiologies of myocarditis and only enrolled adult patients. Moreover, in the cases where viral etiologies may be suspected, an ESC position statement recommends that viral PCR of endomyocardial biopsy be negative before initiation of any immunosuppressive therapy to mitigate risk of exacerbating potential underlying infections (152).

### 4.2 Adjunctive and emerging therapies

The role of IVIG in myocarditis treatment has been explored in both pediatric and adult populations. IVIG has anti-inflammatory, antiviral, and immunomodulatory effects and is relatively safe, with minimal side effects prompting its use, especially when patients present in severe cardiogenic shock with suspected myocarditis. A 2020 Cochrane meta-analysis yielded insights showing no significant improvement in event-free survival or overall survival among adults. There were no changes in LVEF, peak oxygen consumption, or complete recovery rates. Similar findings were reported from the analysis of the single pediatric study (153). However, a separate 2014 study involving 41 patients (aged 19-80) reported improved survival in the IVIG group, though most parameters except lymphocyte and monocyte fractions remained unchanged (154). Another study in pediatric patients with acute encephalitis complicated with myocarditis showed that the use of IVIG was associated with improved LVEF and survival (155). These conflicting results underscore the need for further investigation. There has also been interest in leveraging aspects of fatty acid oxidation. While full mechanisms of action are yet to be understood, trimetazidine is an inhibitor of the long-chain 3-ketoacyl-CoA thiolase, a key enzyme in fatty acid breakdown. Inhibition of this enzyme shifts the primary energy source of the heart from fatty acids to glucose, which leads to lower oxygen consumption and improved myocardial perfusion (156). Combination therapy with Coenzyme Q10 (CoQ10) and Trimetazidine, an inhibitor of fatty acid oxidation, has shown additive benefits in acute viral myocarditis. A 2016 study involving 124 participants demonstrated that CoQ10 and Trimetazidine independently reduced inflammatory markers and improved LVEF. The combination therapy resulted in significantly greater improvements than either treatment alone (157). Additionally, pocapavir, an enteroviral capsid inhibitor, has been shown to be beneficial in treating myocarditis secondary to enterovirus in isolated case reports. However, more extensive studies are still needed (158, 159).

### 4.3 Pediatric-specific treatment studies

Pediatric-specific research highlights unique therapeutic considerations. However, most large-scale randomized control trials for drug approval are conducted predominantly in adult populations, with results being extrapolated to the pediatric population. A 2024 study with 120 children (aged <10 years) compared different doses of creatine phosphate sodium combined with immunoglobulin. The findings revealed that groups receiving higher doses of creatine (1.25g and 1.5g) showed superior therapeutic effects compared to the lowest dose (1g). Significant reductions in biomarkers such as cTnI, CK-MB, LDH, and IL-6, alongside improved LVEF and immune parameters (CD4+/CD8+ ratios), were noted (160).

An additional study in 2023 involving eighty-three children (aged 2–12 years) reported that creatine phosphate sodium reduced heart rate and improved cardiac output, stroke volume, and LVEF within 14 days. The therapy also lowered inflammatory and myocardial injury markers while enhancing immune responses (161).

### 4.4 Clinical trials

Multiple ongoing clinical trials focus on treating myocarditis, which is presented in **Table 2**. The MYTHS trial (Myocarditis Therapy with Steroids; NCT05150704) is focused on treating acute myocarditis with pulsed IV methylprednisolone. In their single-blind randomized trial, pulsed IV steroids will be compared to current standard therapy (162). The ARCHER trial (Impact of CardiolRx on Myocardial Recovery in Patients with Acute Myocarditis; NCT05180240) is a phase II trial focusing on the impact of CardiolRxTM, a cannabidiol preparation, on myocardial recovery in acute myocarditis (163). Interestingly, cannabidiol has been shown to have immunosuppressive effects in both the innate and adaptive systems (164, 165). Additionally, a study in Egypt is recruiting and analyzing the potential benefits of heart rate-lowering therapy in patients with acute myocarditis (NCT06312891). Finally, the ARGO trial (Colchicine Versus Placebo in Acute Myocarditis Patients; NCT05855746) out of France is an ongoing Phase III trial evaluating the impact of colchicine on acute myocarditis. Though multiple trials are ongoing, none have focused their efforts on potential therapeutics considering sex and age as biological variates.

**Table 2:** Current ongoing therapeutic trials in myocarditis. All trials listed are currently being conducted with adult patients.

Trial	Phase	Intervention	Endpoints
Myocarditis Therapy with Steroids (MYTHS)	III	methylprednisolone (1g IV daily for 3 days)	all-cause death, heart transplant, LVAD implant, mechanical circulatory support, VT or Vfib treated with DC shock, or first rehospitalization due to cardiac pathology
Impact of CardiolRx on Myocardial Recovery in Patients with Acute Myocarditis (ARCHER)	II	CardiolRx (Cannabidiol) dose escalation vs placebo Week 1: 2.5 mg/kg BID Week 2: 5 mg/kg BID Week 3: 7.5 mg/kg BID Week 4: 101 mg/kg BID	maximum tolerated dose and analysis of changes on CMR
Colchicine Versus Placebo in Acute Myocarditis Patients (ARGO)	III	Colchicine (0.5 mg twice daily for 6 months) vs placebo	Extent of LGE on CMR and the composite clinical primary outcome
Value of Heart Rate Lowering Therapy in Acute Myocarditis	n/a	Standard therapy vs. standard therapy PLUS carvedilol or Ivabradine	Value of lowering heart rate in short-term cases of myocarditis without LV dysfunction

IV, Intravenous; BID, twice daily; LVAD, Left Ventricular Assist Device; VT, Ventricular Tachycardia; Vfib, Ventricular Fibrillation; DC, direct Current; CMR, Cardiac Magnetic Resonance; LGE, Late Gadolinium Enhancement; LV, Left Ventricle.

### 4.5 Mechanical circulatory support

The use of mechanical circulatory support in the setting of acute myocarditis is relatively low, and guidelines generally reserve these strategies for patients with electrical or hemodynamic instability (90, 166). Short-term mechanical circulatory support is primarily provided using venoarterial membrane oxygenation (VA-ECMO), Impella (usually in >30kg), or implantable devices such as PediMag/Centrimag. Younger patients who require ECMO have a higher mortality risk. However, IVIG administration has been shown to have a protective effect in this population (167–169). In studies specifically looking at children, elevated serum Troponin, female sex, vomiting, seizures, and arrhythmia at presentation may predict the need for ECMO (170, 171). Once initiated, rates of survival and transition off ECMO across studies range from 50% to 70%, which is similar rates seen in studies of adults (172–176).

According to a statement by the AHA, patients who do not recover despite mechanical support transition to a durable ventricular assist device should be considered (177). While rare, recovery of myocardial function with ventricular assist device (VAD) placement can occur, and patients do undergo an explanation of the device in this case (90, 178). However, VAD placement is often used as an effective bridge to eventual cardiac transplant both in pediatrics and adults (179–181). For those patients who do undergo transplant, outcomes for both pediatric and adult patients with myocarditis are similar to those with other cardiomyopathies (182, 183). Conversely, children with clinically or biopsy-diagnosed myocarditis at presentation had significantly higher post-transplantation mortality compared with children without myocarditis. The impact of a history of myocarditis on long-term allograft survival in children may be related to the inflammatory milieu in the individual patients (184).

## 5 Conclusions

Myocarditis is prevalent in both pediatric and adult populations, and the broad spectrum of causes, as well as clinical symptoms, presents a diagnostic difficulty for providers. While there have been great strides in understanding the pathogenesis of this disease, there are still many gaps in understanding. Though future research should continue to investigate the causes of increased risk in adult males, there should also be a stronger focus on research on the pathogenesis of this disease in the pediatric population and, specifically, the differences that may be present between these two populations. As basic and clinical research in this area increases, it may reveal specific mechanisms or biomarkers that may allow for more effective identification and risk stratification in these patients.

Despite advancements in myocarditis treatment, significant gaps remain. Immunosuppressive and anti-inflammatory therapies show variable efficacy, while adjunctive treatments like CoQ10 and Trimetazidine offer promising results. Pediatric-specific studies highlight the importance of tailoring therapies to age-specific needs.

Future research should focus on large-scale, age-stratified RCTs to validate current findings, personalized treatment strategies leveraging biomarkers and advanced imaging techniques, and long-term follow-up studies to assess the durability of therapeutic effects. The evolving understanding of myocarditis pathophysiology offers hope for more effective, targeted therapies in the future.
